# Full-scale numerical simulation of hemodynamics based on left ventricular assist device

**DOI:** 10.3389/fphys.2023.1192610

**Published:** 2023-05-25

**Authors:** Xinyi Gao, Zhike Xu, Chenghan Chen, Pengfei Hao, Feng He, Xiwen Zhang

**Affiliations:** ^1^ Applied Mechanics Laboratory, Department of Engineering Mechanics, Tsinghua University, Beijing, China; ^2^ Tsinghua University (School of Materials Science and Engineering)—AVIC Aerodynamics Research Institute Joint Research Center for Advanced Materials and Anti-Icing, Beijing, China

**Keywords:** heart failure, aorta, full-scale model, hemodynamics, LVAD, CFD

## Abstract

Ventricular assist devices have been widely used and accepted to treat patients with end-stage heart failure. The role of VAD is to improve circulatory dysfunction or temporarily maintain the circulatory status of patients. In order to be closer to the medical practice, a multi-Domain model of the left ventricular coupled axial flow artificial heart was considered to study the effect of its hemodynamics on the aorta. Because whether LVAD itself was connected between the left ventricular apex and the ascending aorta by catheter in the loop was not very important for the analysis of simulation results, on the premise of ensuring the multi-Domain simulation, the simulation data of the import and export ends of LVAD were imported to simplify the model. In this paper, the hemodynamic parameters in the ascending aorta, such as blood flow velocity vector, wall shear stress distribution, vorticity current intensity, vorticity flow generation, etc., have been calculated. The numerical conclusion of this study showed the vorticity intensity under LVAD was significantly higher than that under patients’ conditions and the overall condition is similar to that of a healthy ventricular spin, which can improve heart failure patients’ condition while minimizing other pitfalls. In addition, high velocity blood flow during left ventricular assist surgery is mainly concentrated near the lining of the ascending aorta lumen. What’s more, the paper proposes to use Q criterion to determine the generation of vorticity flow. The Q criterion of LVAD is much higher than that of patients with heart failure, and the closer the LVAD is to the wall of the ascending aorta, the greater the Q criterion is. All these are beneficial to the effectiveness of LVAD in the treatment of heart failure patients and provide clinical suggestions for the LVAD implantation in clinical practice.

## 1 Introduction

Heart failure is a serious stage of cardiac function damage caused by various cardiovascular diseases and related diseases, with high incidence and fatality rate (Maeda et al., 2023). It has become one of the most important cardiovascular diseases in the 21st century. For the early stage of heart failure, symptoms can be alleviated by injecting drugs to ensure fluid stability. Diuretics can be used to reduce blood volume and thus reduce the burden on the heart (van Poelgeest et al., 2023). But prolonged use will lead to diuretic resistance, which reduces the effectiveness and requires continuous injection to maintain high levels of the drug. Vasodilators can dilate blood vessels and reduce resistance to blood flow, thus effectively treating heart failure caused by dilated cardiomyopathy, ischemic heart disease, and valve insufficiency (Siems et al., 2023). However, vasodilators can cause certain low blood pressure, which will lead to ischemia in the brain. With the development of drugs for treating heart failure, heart transplantation technology and ventricular assist technology are also developing simultaneously in China. The in-hospital fatality rate of heart failure patients has an obvious downward trend (Cogswell et al., 2020). At present, although heart transplantation is still the best treatment for patients with end-stage heart failure, problems such as donor heart shortage, in vitro heart preservation and immune rejection after transplantation cannot be ignored (Crescioli, 2013). Therefore, mechanical circulatory assistance devices, represented by ventricular assist devices and artificial hearts, have gradually become the preferred method in the transitional period of heart transplantation (Pagani, 2020), and may even become the alternative method for long-term treatment. LVAD has become an important treatment method for patients with heart failure (Chahal et al., 2019). LVAD is a blood pump that can provide power for blood circulation. According to different types of pumps, it can be divided into two categories: pulsation type and continuous type. Pulsation type works like a heart, with a one-way valve that controls blood flow and compresses and dilates chambers to transport blood, so it can synchronize heart beats to a certain extent (Sun et al., 2020). Its therapeutic purposes mainly include: as a pre-transplant transitional support therapy, permanent supportive therapy, pre-recovery transition, pre-decision transition and transition-transition therapy. The current clinical research focus of LVAD has shifted to permanent supportive therapy. LVAD can not only reduce the preload and afterload of the heart, but also the oxygen consumption of the myocardium. Increasing the diastolic blood pressure, and the blood flow of coronary artery circulation. What the LVAD do is promoting collateral circulation, and further improving the contractility of myocardium; promoting the enhancement of cardiac pump function, and accelerating the recovery of myocardium. LVAD can even replace the cardiac pump function before surgical treatment (including heart transplantation). According to the 2020 annual report of the Registry of Mechanically Assisted Circulation Support Institutions (INTERMACS) database (Cogswell et al., 2021), a total of 3,198 patients in the United States received LVADs in 2019, of which 73% received LVADs as permanent support therapy and 77% received LVADs featuring magnetic levitation technology. The 1-year and 2-year survival rates of patients supported by LVAD were 82.3% and 73.1%, respectively. The 1-year survival rates were close to those of heart transplant patients, indicating the importance of LVAD assisted treatment for patients with heart failure.

Over the past few decades, computational fluid dynamics (CFD) technology has made remarkable progress. Compared with experimental methods, CFD can provide more details of the flow field, enabling researchers to analyze the flow mechanism in fluid machinery (Tominaga and Stathopoulos, 2013). Carswe (Carswell et al., 2013) et al. proposed a computational model based on CFD method to predict hemolysis in micro-LVAD. Zhang (Liu et al., 2016) et al. developed a series of axial blood pumps, which effectively improved the blood compatibility of blood pumps by optimizing the flow structure and eliminating the irregular flow structure in the flow field. Thamsen (Thamsen et al., 2015) et al. use the working states of HeartMate II and HeartWare HVAD blood pumps were numerically solved by dynamic grid method, and the fluid volumes corresponding to different shear forces were calculated. It was found that the volume of the two blood pumps with shear force greater than 10Pa was basically the same, so it was concluded that the two blood pumps had similar hemolysis index. Schule (Schule et al., 2016) et al. used HeartMate II prototype, 3:1 model and numerical calculation for research, observed the flow field through Wall-PIV, and compared with the numerical simulation, found that the SST k-omega RANS turbulence model can reliably predict the flow field, and the calculated wall shear force has a good consistency with the experiment. There have been sufficient studies on patients with heart failure under the support of LVAD, most of which focus on the hemodynamics and pathophysiology differences under the support of LVAD. However, these studies focus on the influence of LVAD on aortic hemodynamics. Due to the complexity of the model, the possible influence of left ventricle on aortic hemodynamics is ignored. By constructing a multi-Domain model of the left ventricular coupled axial flow artificial heart, studies on the hemodynamic effects on the aorta are still incomplete.

How to effectively address the disease in heart failure patients by altering hemodynamic parameters. It is worth noting that the whirling of blood flow in the aorta is a high manifestation of nature’s unity of form and function (Kalluri et al., 2019) and a guarantee of smooth scour of the aortic vascular wall to prevent the formation of atherosclerosis. Normally, flow separation is easily caused by factors such as pulsation of blood flow and curved shape of blood vessels, and one of the main functions of vorticity flow is to eliminate or weaken flow separation and disorder (Candel et al., 2014). Its tangential velocity sweeps along the medial wall of the aorta to prevent fluid separation from the medial wall due to forward momentum. Secondly, vorticity flow can significantly improve the magnitude and distribution of wall shear force, and better inhibit the occurrence of atherosclerosis, intima hyperplasia, thrombus adhesion and other diseases (Charlesworth and Gerrard, 1988). In addition, vorticity can increase blood perfusion to bifurcated vessels, such as during ventricular diastole, when blood flow is slowed down or even partial reflux effect occurs. The convective acceleration to maintain the effective perfusion of bifurcated vessels is likely to be provided by the vorticity flow at the bifurcation. Although the blood flow velocity is slow during diastole, the flow radius of the local whirling flow is much smaller than the curvature radius of the aortic arch. Therefore, a larger convective acceleration will also be generated, thus maintaining a larger perfusion pressure on the tip of the aortic arch (Goto et al., 2021). The latest research results of Watkins (Watkins et al., 2002) et al. show that whirling flow can increase the transmission of oxygen in the blood to the arterial wall. Meanwhile, according to Deng (Zhan et al., 2014) et al. ‘s concentration polarization theory, the vorticity flow pattern also affects the deposition rate of atherosclerotic lipids (such as LDL, *etc.*) in the vessel wall. Deng, Liu (Zhan et al., 2014) et al. analyzed in detail the influence of the vorticity flow pattern in the aorta on LDL and oxygen transport by numerical simulation. The vorticity flow could inhibit the deposition of atherosclerotic lipids (LDL) to the arterial wall, and at the same time increase the oxygen transport to the arterial wall. Studies show that vorticity flow is an important physiological flow phenomenon, and there are many related scientific researches. It was found that the vorticity flow can improve the uniformity of wall shear stress, reduce the stagnation of flow, reduce the surface concentration of low-density lipoprotein, and increase the oxygen flow (Zhang et al., 2022). Since human endovascular vorticity flow is considered a classic example of “form-following function,” which is generally considered necessary to maintain the good functioning of the aorta, there has been a lot of research on it (Raghav et al., 2018). However, it is found that there are no studies and analyses on the influence of LVAD support on hemodynamic and vorticity flow characteristics related to multi-Domain aortic models.

Based on the CT data of patients with heart failure, LVAD was connected between the left ventricular apex and the ascending aorta via catheter to reconstruct the specific geometric model of aorta in patients with heart failure (Zuo et al., 2022). Although LVAD intervention affects more the changes in hemodynamic parameters of the aorta connected to its posterior end, since whether LVAD intervention affects the original hemodynamic parameters of the left ventricle will bring many influencing factors in medical work, we should make the access LVAD as small as possible to trigger other induced combined diseases. In view of this we believe it is necessary to construct a multi-Domain LV model when constructing the model, i.e., including four parts: LV, aorta, pipe, and LVAD. In this way, by observing the changes in the hemodynamic parameters of the intervening LVAD in heart failure patients and the healthy human body as a whole, we can better assist patients and reduce the chance of induced disease.

Using Computational Fluid Dynamics (CFD) to simulate the left ventricular coupled axial flow artificial heart (named LVAD case), heart failure patients (named heart failure case) and healthy people (named healthy case) at multi-Domain. The blood flow patterns and characteristics of the aorta were studied, and the hemodynamic parameters in the ascending aorta were calculated, such as the flow velocity vector, wall shear stress (WSS) distribution, vorticity current intensity. And using the Q criterion to determine whether the vorticity flow state was generated. To evaluate the treatment status of LVAD in patients with heart failure, so as to provide theoretical guidance and clinical recommendations for clinical implantation of VADs.

## 2 Materials and methods

### 2.1 Geometric model reconstruction

**FIGURE 1:**
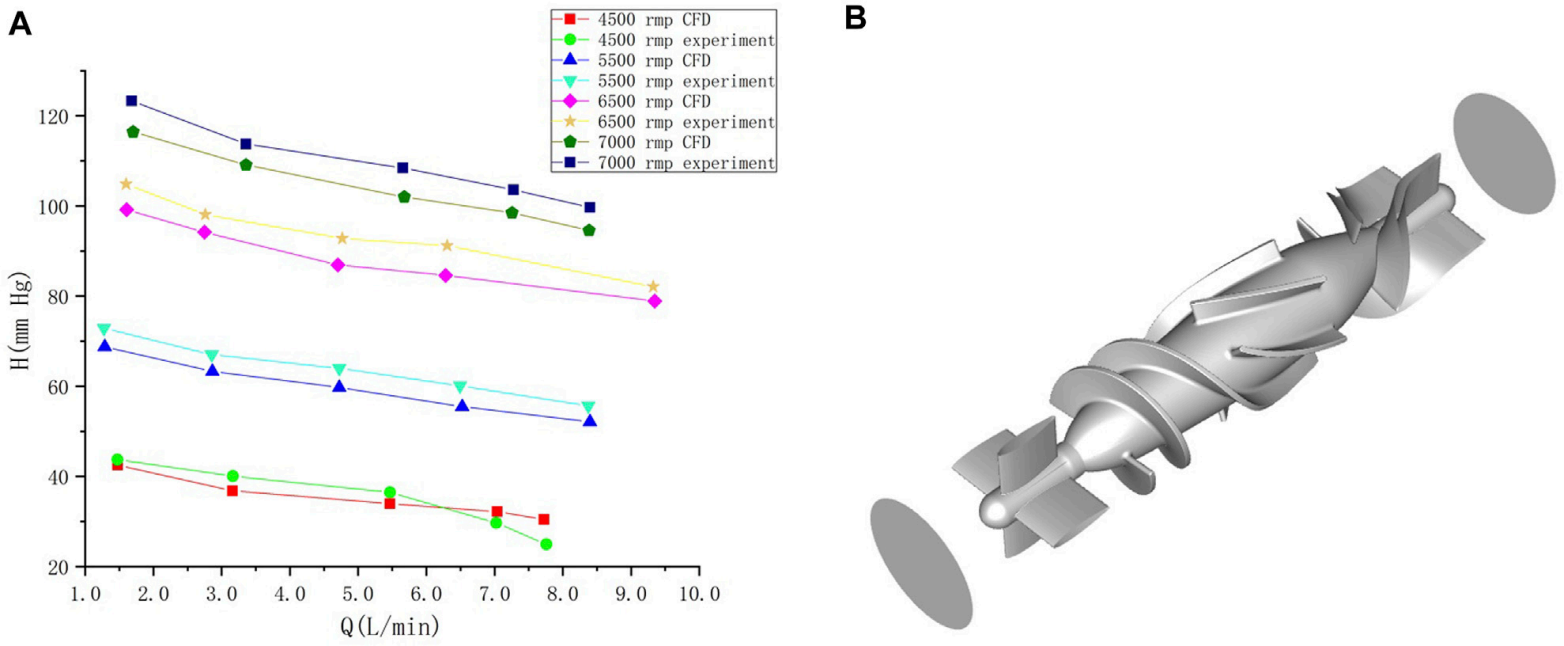
**(A)** Comparison of calculated results and experimental results under different rotational speeds of the blood pump. Abscissa Q is the flow rate at the outlet after the pump, and ordinate H is the pressure at the outlet after the pump (Wu, 2016). **(B)** blood pump and the inlet/outlet of the blood pump.

**FIGURE 2:**
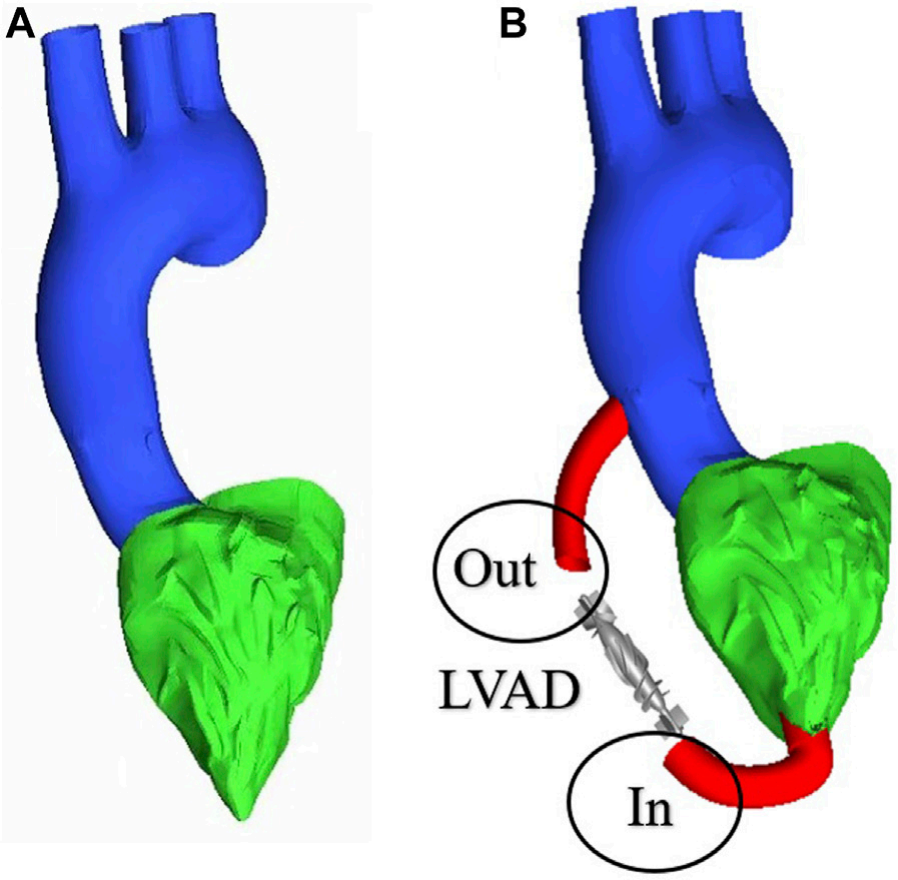
Multi-Domain geometric model **(A)** heart failure patient case/healthy human case **(B)** LVAD case.

To obtain changes in hemodynamic status, a series of computed tomography angiography (CTA) images were used to reconstruct multi-Domain models of the left ventricular coupled axial-flow artificial heart (named LVAD case) and heart failure patient (named heart failure case), the ethical requirements were obtained in the previous publication (Zuo et al., 2022). The specific 3D model of the aorta was reconstructed using these images and commercial 3D reconstruction software MIMICS (Materialise, Belgium). And then the model was then fed into the Geomagic (Geomagic, United States) software to improve the surface quality of the aorta. Our experimental CT data were obtained from Chang Gung Hospital of Tsinghua University (Beijing, China), it is part of a routine check-up and the model was extracted from a clinical heart failure patient (60–70 years old). Since the structure of CT scans of real heart failure patients is overly complex, which will lead to a confusing grid structure and is not suitable for subsequent simulations, here we made a structural repair based on the existing model to improve the grid quality, and we consider here the use of the “classical heart model”. We use a continuous blood pump here. Pulsatile blood pumps mimic the pulsation of the heart and can produce a regular pulsatile flow, thus better meeting the needs of ventricular unloading and avoiding the phenomenon of ventricular pumping. However, due to the excessive size of the pulsating blood pump, it makes great demands on the installation and places a great burden on the body. It can also compress organs and even cause infections. Continuous type blood pumps are relatively small and less burdensome to the body, and have been shown to Continuous flow has been shown to cause limited damage to the body, and therefore has become the mainstream of blood pump research. The LVAD axial blood pump used in this study was designed by the Department of Engineering Mechanics of Tsinghua University (Beijing, China), including flow straightener, impeller (rotating blade) and diffuser, with a length of 72 mm and an outer diameter of 16 mm. The clearance between the rotor blade and the shell is 0.1 mm. The front guide blade and the rear guide blade are evenly distributed with 4 blades. The rotor blade is a combination of two main blades and four auxiliary blades. The pump is an external magnetic driven pump, equipped with a pair of ceramic bearings, the outer diameter of the pump shell is 20 mm, the inner diameter is 18 mm, the hub diameter is 10mm, and the axial clearance between the impeller and the air flow straightener is 2 mm. Wu (Chen et al., 2013) et al. have demonstrated that the experimental flow performance of the blood pump is consistent with the CFD prediction results. The results of LVAD in the whole speed range are in good situation, and the outlet pressure decreases with the increase of the flow rate, which conforms to the principle of energy conservation. The numerical results of the ventricular assist device are in good situation with the measured results in the whole speed range (Figure 1A). Due to the complexity of the mesh of the blood pump itself, adding the whole blood pump into the model will greatly increase the mesh complexity. It is difficult to obtain excellent mesh quality for subsequent calculation. Therefore, in this study, the numerical simulation velocity section of the blood pump inlet and outlet was introduced at both ends of the catheter inlet and outlet. The relevant parameters of the numerical simulation of the blood pump were briefly introduced here (Chen et al., 2013): 2.04 million grid was adopted, the rotation speed was 7,000 rpm, the maximum speed of the inlet section was 0.031640 kg/s, and the maximum speed of the outlet section was 0.031632 kg/s (Figure 1B). Then ICEM 2021 (ANSYS, Inc., United States) was used to construct multi-Domain geometric models of left ventricular coupled axial flow artificial heart (LVAD case) and heart failure patient heart/healthy heart (heart failure case/healthy case) (Figure 2). For the common part of Figures 2A, B: the green area represents the ventricular model and the blue area represents the aortic model. Due to the complexity of the human whole ventricle structure, we chose to construct almost identical multi-Domain geometric models of heart failure patients and healthy humans. The distinction between heart failure patients and healthy humans was made by varying the blood flow rate at the ventricular inlet. Here we set the left ventricular inlet mass flow rate to 4.5 L/min in healthy human case and 2.5 L/min in heart failure patients case. The intervention of the blood pump will increase the complexity of the mesh division and seriously affect the quality of the mesh. The thickness of the intervening tube in our LVAD arithmetic model is exactly the outer diameter of the LVAD. So we use the velocity of the LVAD entrance/exit section to be exported and then import the velocity of each node into the LVAD arithmetic entrance/exit section. For Figure 2B, the red area represents the artificially added pipes, and the grey area is the LVAD intervention area (For ease of understanding we show the location of LVAD in Figure 2, due to the complexity of the LVAD model, in practice we did not intervene in the full LVAD model, but used to import the entrance and exit section velocity) Since the LVAD intervention has a large impact on the mesh complexity, we adopt here to import the LVAD existing exit and entrance velocity cross-sections. Since the variability between the heart failure patient model and the healthy human model in our model construction is mainly reflected in the ventricular inlet flow, the model differences are not significant, so the healthy human heart model is not repeatedly shown here.

### 2.2 Mesh generation

**FIGURE 3:**
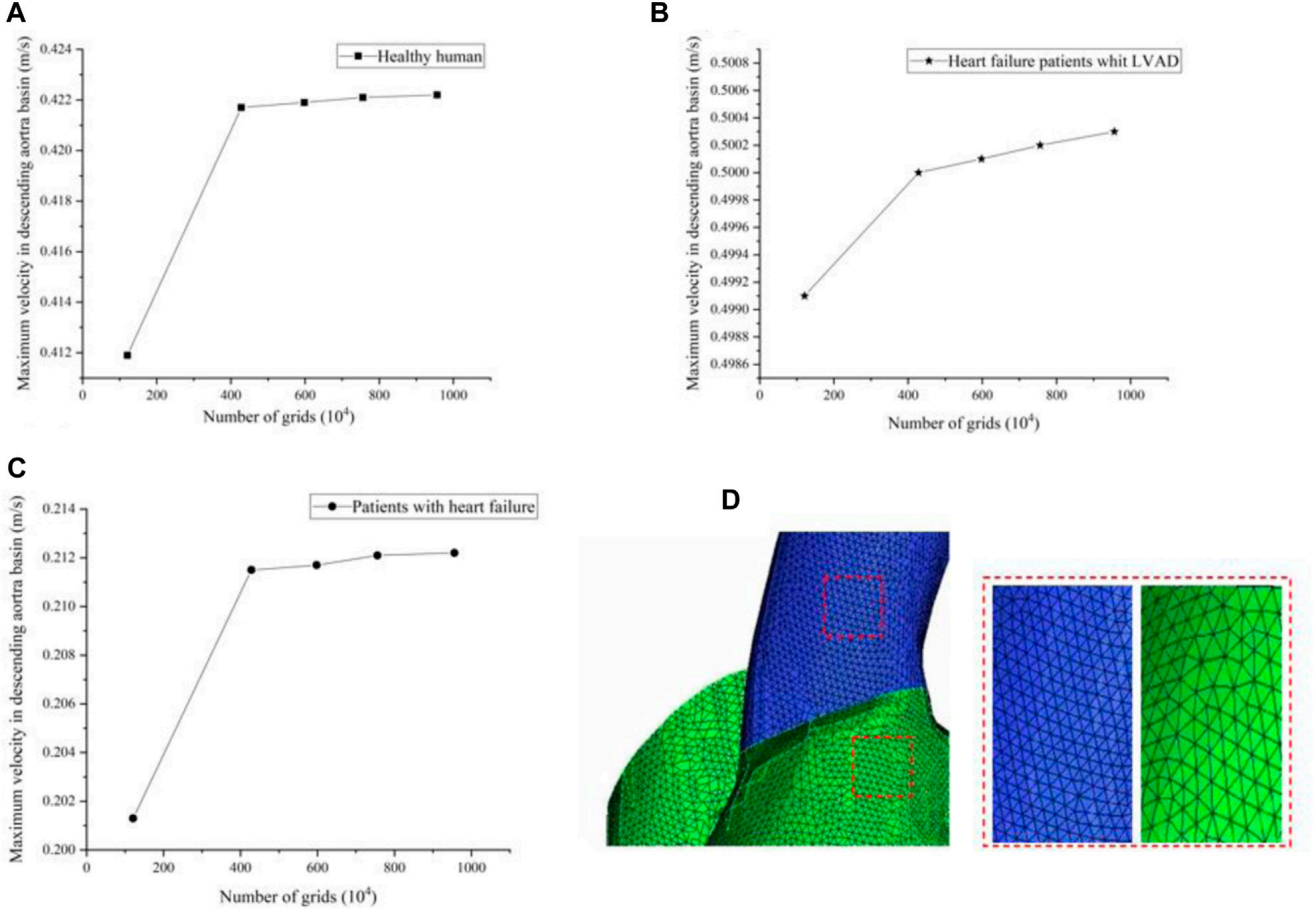
**(A–C)** The maximum velocity of the ascending aortic basin in different grids **(D)** Meshing in this study.

In this study, grid generation tools (ICEM CFD, ANSYS, Inc., United States) were used for grid division of LVAD cases, 1.21 million, 4.28 million, 5.98 million, 7.56 million and 9.56 million grids were selected for grid sensitivity analysis. Under the same condition of left ventricular inlet mass flow (4.5 L/min), and left ventricular inlet mass flow (2.5 L/min) in patients with heart failure, observe the change of maximum velocity in ascending aorta basin with the number of grids. We found that with the increase of grid number, for healthy humans the maximum velocity of the ascending aortic basin is respectively 0.4119 m/s, 0.4217 m/s, 0.4219 m/s, 0.4221 m/s, 0.4222 m/s (Figure a)), for patients with heart failure the maximum velocity of the ascending aortic basin is respectively 0.2013 m/s, 0.2115 m/s, 0.2117 m/s, 0.2121 m/s, 0.2122 m/s (Figure b)). Restate the above steps for heart failure patient’s whit the LVAD intervention, whose left ventricular inlet mass flow is 2.5 L/min. The maximum velocity of the ascending aortic basin is respectively 0.4991 m/s, 0.5001 m/s, 0.5004 m/s, 0.5006 m/s, 0.5007 m/s (Figure c)). We found that the error caused by the grid is within 1%. Under the premise of not losing too much precision and ensuring computational efficiency, 4.28 million grids were selected for calculation in this experiment. Figure 3D shows the meshing in this study.

### 2.3 Numerical approaches and calculation settings

In this study, blood is assumed to be an incompressible uniform Newtonian fluid. The flow simulation is based on the conservation of momentum and mass in incompressible fluids (Zhang et al., 2020), known as the Navier-Stokes equations.

$$\begin{array}{c} \nabla \cdot u=0\\ \rho \frac{\partial u}{\partial t}+\rho \left(u\cdot \nabla \right)u=-\nabla p+\mu_{t}\nabla^{2}u \end{array}$$ (2.1)

Let u as the velocity vector, t for time, p for pressure, ρ and $\mu_{t}$ for the density of blood and turbulent viscosity. In this study, the blood was assumed to be a uniform incompressible Newtonian fluid with a density of 1,050 kg/m^3^ and a viscosity of 0.0035 Pa s (Cho et al., 2014), respectively, and the aortic wall was assumed to be non-slip rigid. The simulation was carried out under static flow conditions. The inlet volumetric flow of the healthy human was set as 4.5 L/min and 2.5 L/min with patients with heart failure (Kim and Hong, 2019). All the outlet conditions of the model were zero pressure boundary conditions. In addition, the LVAD’s speed is set to 7,000 rpm and the rotation direction is counterclockwise (viewed from the inlet direction). In addition, the convergence accuracy of residuals in this study is set at 10^–3^. The zero-pressure boundary condition is chosen as the exit boundary condition. Because the deformation of aortic wall morphology has little effect on blood flow morphology, we ignored the deformation of aortic wall here. The pattern of blood flow in the aorta is mainly determined by the pressure gradient. Therefore, it has little influence on the accuracy by the results of zero pressure boundary conditions. These basic equations were solved using the finite-volume CFD solver ANSYS Fluent 2020. The simulation used the semi-implicit pressure correction link equation based on pressure correction (SIMPLE algorithm) to solve the N-S equation. Monitoring of aortic velocity flow to stabilize at a certain interval of fluctuation when the calculation is considered converged and the average results of the last 200 steps are counted.

### 2.4 k- ω SST turbulence model

When solving the turbulent flow problem, the solution variables in the N-S equation will be decomposed into the mean value and the wave component. Velocity component $u_{i}$ also broken down into average velocities ${\bar{u}}_{i}$ and perturbation velocity $u_{i}={\bar{u}}_{i}+u_{i}^{\prime }$ **.** By substituting it into the ordinary N-S equations, the Reynolds mean N-S equations (RANS) can be obtained (Vassilicos, 2015):

$$\frac{\partial \left(\rho u_{i}\right)}{\partial t}+\frac{\partial \left(\rho u_{i}u_{j}\right)}{\partial x_{i}}=-\frac{\partial p}{\partial x_{i}}+\frac{\partial }{\partial x_{j}}\left(\mu \left(\frac{\partial u_{i}}{\partial x_{j}}+\frac{\partial u_{j}}{\partial x_{i}}\right)\right)+\frac{\partial }{\partial x_{j}}(-\rho \overset{\_\_\_\_\_}{u_{i}^{\prime }u_{j}^{\prime }})$$ (2.2)

Compared with the ordinary N-S equations, the Reynolds stress term is generated in the momentum equation for the RANS equations due to the turbulence component $-\rho \overset{\_\_\_\_\_}{u_{i}^{\prime }u_{j}^{\prime }}$ . The RANS must be sealed by building turbulence models and adding additional equations (Wang et al., 2021). Common turbulence models include k−ε (turbulent kinetic energy - dissipation rate) model, k−ω (turbulent kinetic energy - specific dissipation rate) model, *etc.* From the geometric size, blood density and blood flow velocity of the multi-Domain model, the peak Reynolds number Re is greater than 5,000 (Freund, 2014). Therefore, the k-ω SST turbulence model is used in this study, which gradually changes the standard equation model in the inner region of the boundary layer into the high Reynolds number equation model outside the boundary layer by using the mixing function. The vorticity current viscosity formula is modified, which can accurately predict the start of flow and the amount of fluid separation under the negative pressure gradient. The most important advantage of this model is that the turbulence shear stress is taken into account, so as not to overpredict the vorticity viscosity. The turbulence model is proved to be suitable for describing the turbulence flow state of the multi-Domain LVAD model. The model assumes that turbulence viscosity is related to turbulence kinetic energy, turbulence frequency or specific dissipation ω:

$$\mu_{t}=\rho \frac{k}{\omega }$$ (2.3)

Turbulent kinetic energy **k** and specific dissipation rate ω transport equation is as follows (Racina and Kind, 2006):

$$\rho \frac{\partial k}{\partial t}+\rho U_{j}\frac{\partial k}{\partial x_{j}}=\tau_{ij}\frac{\partial U_{i}}{\partial x_{j}}-\beta^{*}\rho k\omega +\frac{\partial }{\partial x_{j}}\left[\left(\mu +\sigma^{*}\mu_{t}\right)\frac{\partial k}{\partial x_{j}}\right]$$ (2.4)

$$\rho \frac{\partial \omega }{\partial t}+\rho U_{j}\frac{\partial \omega }{\partial x_{j}}=\alpha \frac{\omega }{k}\tau_{ij}\frac{\partial U_{i}}{\partial x_{j}}-\beta \rho \omega^{2}+\frac{\partial }{\partial x_{j}}\left[\left(\mu +\sigma_{\omega }\mu_{t}\right)\frac{\partial \omega }{\partial x_{j}}\right]+2\left(1-F_{1}\right)\rho \sigma_{\omega }\frac{\partial k\partial \omega }{\omega \partial x_{j}\partial x_{j}}$$ (2.5)

### 2.4 Vortex volume calculation

Vortex identification is a common method to analyze flow structure in fluid mechanics. It can reflect the three-dimensional vortex structure in flow field effectively. Among them, vortex recognition method based on velocity gradient tensor has been proved to be an effective and widely used tool for vortex structure recognition. Perry and Chong proposed ∆ method, which defines vortices as regions where the eigenvalues of the velocity gradient tensor are complex numbers. In order to more clearly judge the vortex structure of the case of heart failure patients and the case of LVAD, and the case is considered as incompressible fluid, Q method is adopted as the criterion. That is, Q > 0, Q represents the balance between shear strain rate and vorticity size (Arshad et al., 2021).

The accuracy of the vortex calculation is decisive for the identification of vortices. Vortex quantities are used to represent the intensity of rotating fluid motion, and visual presentation through parametric fields alone is usually insufficient to describe the rotation of the fluid (vorticity flow). Although such velocity information is limited to a concise description of the flow, the use of additional quantities helps to gain a deeper understanding of the fluid motion. Therefore, based on the unique properties of the full-centered chamber flow field and the theoretical basis of finite element analysis, an equation for the calculation of vorticity is derived for the full-centered chamber flow field. The Vorticity density (Ha) (in s^-1^) is defined as the spin of the fluid velocity V, where:

ω=∇×v (2.6)

It is a representation of the local direction of rotation and angular velocity used to indicate the vortex core and its rotation. The strength of a vortex can be characterized by analyzing the vortex volume field of the vortex. From a hydrodynamic point of view, the velocity flow field alone is not sufficient to create a complete description of the flow. Other metrics can be computed in the flow field that will better describe the state of flow field motion. If resolved in time, these metrics can be used to study the flow properties of the flow field. A prerequisite for calculating the flow parameters is the finite element discretization technique, which uses a suitable window for sampling the data to facilitate the calculation of the velocity field discretization.

$${\left(\frac{\partial f}{\partial x}\right)}_{i}=\frac{1}{N}\sum_{n=1}^{N}f_{i}^{\prime }\left(2\Delta x\right)\approx \frac{1}{N}\sum_{n=1}^{N}\frac{f_{i+1}-f_{i-1}}{2\Delta x}$$ (2.7)

Using the above equations, the differential components of the flow field velocity in the *x* and *y* directions are calculated. The vorticity is calculated from the velocity field of the blood flow in the heart cavity and is the line integral of the tangential velocity around a point along a loop. The vorticity is equal to the loop flow divided by the area enclosed by this loop is equal to the loop flow divided by the area enclosed by this loop. The vortex volume is calculated based on the velocity rotation at a point in the flow field and is calculated as

$$\omega ={\left(\frac{\partial V_{y}}{\partial V_{x}}-\frac{\partial V_{x}}{\partial V_{y}}\right)}_{i,j}=\lim_{\Delta x,\Delta y\to 0}\;\frac{1}{N^{2}}\sum_{m=1}^{N}\sum_{n=1}^{N}\left[\begin{array}{c} \frac{V_{y}\left(i+m,j+n\right)-V_{y}\left(i-m,j+n\right)}{2m\Delta x}-\\ \frac{V_{x}\left(i+m,j+n\right)-V_{x}\left(i+m,j-n\right)}{2n\Delta y} \end{array}\right]$$ (2.8)

In the formula, a positive value indicates counterclockwise rotation, while a negative value indicates clockwise rotation of the blood fluid. Thus, the magnitude of the value indicates the rate of rotation, while its polarity indicates the direction of rotation.

## 3 Results and discussions

### 3.1 Hemodynamic parameters based on LVAD

**FIGURE 4:**
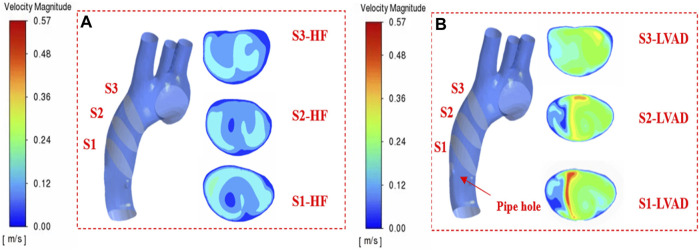
S1, S2, S3 cross section velocity contour **(A)** heart failure patient case **(B)** LVAD case.

In order to evaluate the effect of multi-Domain LVAD model on the hemodynamics of ascending aorta in patients with heart failure, we compared the case of LVAD with the case of heart failure, and discussed the flow line, blood flow velocity, blood flow vector, mean Vorticity density, WSS and other related parameters, as well as whether the vorticity state was generated. Through literature review, we found that the cardiac inlet volume flow in normal human body is about 5 L/min, and the mass flow in ascending aorta is about 0.08 kg/s (Arshad et al., 2021; Chen et al., 1156). In order to verify whether the numerical simulation has practical medical significance and whether it can ensure the practical effect of the blood pump at a rotational speed of 7,000 rpm, three representative sections were selected longitudinally along the central line of the aorta in this study, as shown in Figure 4.

In patients with heart failure, there is an obvious low-velocity region in the middle region of the aorta in the shape of a circle, while in the case of LVAD, the obvious high-speed region is in long strip, and the high-speed region is close to the inner wall of the aorta (Figure 4 S1, S2-LVAD). Three cross-sectional locations selected with reference to medical materials (Racina and Kind, 2006), section 1(S1) was located anterior to the ascending aorta. Section 2(S2) was located in the middle of the ascending aorta. Section 3(S3) was located posterior to the ascending aorta. The maximum speed on S1 section is 0.55 m/s. Due to the cross-section area of the connecting pipe outlet is 159 mm^2^. We found that mass flow in the ascending aorta of patients with heart failure treated by LVAD through numerical simulation can reach 0.087 kg/s, which is consistent with the results of medical experiments (Yao et al., 2021). As the LVAD intervention drove blood from the tube outlet into the aorta, it made a low velocity flow zone in the aorta near the tube outlet side region. Due to the model that completely simulated the ventricle and the aorta, there was a valve at the junction of the ventricle and the aorta, resulting in the ascending aortic jet being divided into two parts. The low velocity jet near the pipe and the high velocity jet near the medial side. From the velocity contour in Figure 4B, it can be seen that there was a flow separation zone at the LVAD intervention tube and aortic junction, which drove aortic blood from the ascending aorta as the LVAD rotates at high speed and gradually formed a jet deeper into the ascending aorta. The flow separation zone is gradually shifted upward and to the right from the insertion site and the degree of separation decreases, which is due to the gradual decrease in the degree of jet formed by LVAD intervention and the average area. The effect of which is similar to the blood flow in the real healthy human ascending aorta with less difference in flow field distribution. At the same time, vorticity flow was formed under the action of LVAD high-speed jets, and the formation of vorticity flow helped to reduce the low-flow velocity zone and stagnation zone within the blood chamber, thus improving the intravascular surface flushing effect and reducing the risk of thrombus formation. As the effect of LVAD diminished, the intensity of the vorticity flow gradually decreased, and the velocity of the flow field in the upper part of the ascending aorta decreased, and the high-flow velocity area was mainly concentrated in the inner curvature of the ascending aorta, which was also consistent with the real healthy ventricle situation (Arshad et al., 2021).

**FIGURE 5:**
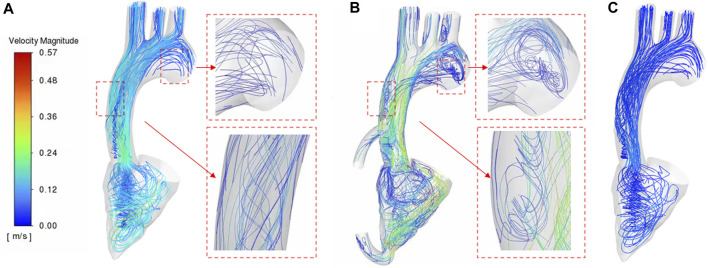
Blood streamline **(A)** healthy case **(B)** LVAD case **(C)** heart failure patient case.

As can be seen from Figure 5, the blood flow of LVAD patients is significantly higher than that of patients with heart failure (LVAD patients 0.57 m/s, heart failure patients 0.28 m/s). In addition, there were significant differences in the flow patterns in the aorta between the LVAD and heart failure cases. Compared with the case of heart failure, in the LVAD case, obvious counterclockwise vorticity flow was observed near the ascending aorta and in the aortic arch. The vorticity direction was composed of the rotation direction of the left ventricular assist device connected in this experiment, and the vorticity direction was close to the aortic wall. This is caused by the LVAD intervention generating a jet at the pipe and ascending aorta, which is a high velocity jet, occupied by a vorticity flow, and the wall of this vorticity flow is a high flow velocity zone, which happened to form a good washout of the ascending aorta, increasing the wall shear rate there, decreasing the probability of platelet deposition. Thus reducing the risk of thrombosis. In addition, the presence of a valve between the ventricle and the aorta results in a high flow rate zone, where high flow rates produced high shear, which increased the destruction of red blood cells and platelet activation (Bermejo et al., 2014). It increased the risk of hemolysis and thrombosis.

**FIGURE 6:**
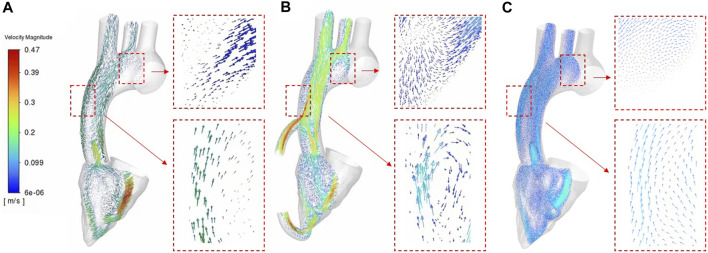
Velocity vector contours **(A)** healthy case **(B)** LVAD case **(C)** heart failure patient case.

In order to clearly show the velocity fields of the two cases, this study intercepted the cross sections along the central axis of the aorta. We discussed the velocity profiles vectors of the three cases on the same cross section. Figure 6 shows the flow velocity vector distribution contours of three cases. Figure 6A shows the cross-section flow velocity vector contour of healthy human. Figure 6B shows the cross-section flow velocity vector contour of LVAD case. Figure 6C shows the cross-section flow velocity vector contour of patients with heart failure. As can be seen from Figure 6, the blood flow pattern under LVAD is very different from that in patients with heart failure. Because LVAD rotation drives rapid blood flow, obvious high-speed bands can be seen at the outlet nozzle in the case shown in Figure 6B LVAD. The maximum velocity reached 0.43 m/s. On the aorta on the other side of the tube, also due to LVAD, the wall velocity in the case of LVAD (Figure 6B, 0.25 m/s) was higher than that in the case of healthy human (Figure 6A, 0.069 m/s). It was found in the study that obvious low-velocity region was visible in the ascending aorta in the case of LVAD above the takeover (Figure 6B, 0.069 m/s). This is different from the distribution of blood flow velocity in the ascending aorta of healthy human (Figure 6A, 0.26 m/s), showing a stagnation zone. In addition, the overall blood flow velocity in patients with LVAD (Figure 6B, 0.28 m/s) was significantly higher than that in healthy human (Figure 6A, 0.11 m/s) farther from the aortic arch, and the area with high blood flow velocity was located in the inner wall of the aorta. By observing the cross-section blood flow velocity vector diagram, obvious vorticity flow was generated at the ascending aorta in the case of LVAD above the tube, while no such phenomenon was found in the case of heart failure patients, presenting an upward flow velocity vector diagram. In the lower part of the medial wall of the aortic arch, vorticity flow was observed in the LVAD case, but not in the heart failure case, presenting a velocity vector diagram of oblique upward flow to the right. The numerical conclusion of this study showed that LVAD connected by catheter between the left ventricular apex and the ascending aorta could significantly change the blood flow pattern, vorticity flow characteristics and WSS distribution in the ascending aorta, and the vorticity intensity under LVAD was significantly higher than that under normal conditions. In addition, high velocity blood flow during left ventricular assist surgery is mainly concentrated near the lining of the ascending aorta lumen. Under normal conditions, high-speed blood flow is mainly concentrated in the central region of the aortic lumen. All these are beneficial to the effectiveness of LVAD in the treatment of heart failure patients.

In addition, vorticity flow reduces the concentration of low-density lipoprotein (LDL) on the lumen surface of the aortic arch, inhibit severe atherosclerosis, and regulate the function of vascular smooth muscle cells. This study showed that the strength of the cyclone was significantly enhanced after LVAD support (Figure 6), indicating that the cyclone generation could be promoted by connecting the LVAD to the ascending aorta with an external catheter from the left ventricular apex. As can be seen from Figure 5B, the rotation direction of blood flow under the case of LVAD is counter clockwise, which is consistent with the rotation direction of LVAD. However, under normal conditions, the vorticity flow generated by aorta is mainly clockwise. According to Chien’s findings (Zhang et al., 2022), flow patterns can determine the arrangement and characterization of endothelial cells. Therefore, the change in the rotation direction of the internal aortic vorticity flow may lead to aortic remodelling and thus reduce the symptoms of heart failure. The specific effect needs to be further studied. The high blood flow velocity region in the LVAD case was located near the ascending aorta wall (Figure 6B), while the high blood flow velocity region in the heart failure case was located in the canter of the aortic lumen (Figure 6C). This means that atherosclerotic plaque and LDL are less likely to be deposited on the Ascending aorta wall than in patients with heart failure under LVAD due to high blood flow velocity.

**FIGURE 7:**
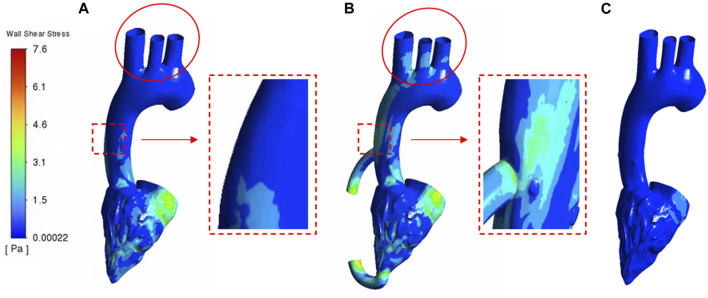
WSS cloud contours **(A)** healthy case **(B)** LVAD case **(C)** heart failure patient case.

Figure 7 shows the WSS cloud map of the calculation cases of heart failure patients and LVAD patients. In general, WSS intensity was significantly higher in LVAD than in heart failure (maximum 7.6 Pa in LVAD and 0.6 Pa in heart failure). The higher WSS in LVAD was concentrated in the ascending aorta (Figure 7B, red square, 4.7 Pa) and aortic arch (Figure 7B, red circle, 4.2 Pa). In addition, patients with heart failure had very low WSS in the aortic arch (Figure 7A, red circle, 0.02 Pa). The results showed that WSS of ascending aorta in LVAD was significantly higher than that in heart failure. It was found that WSS plays an important role in regulating the arrangement and function of endothelial cells. Chakraborty et al. (Chakraborty et al., 2016)demonstrated that WSS is an important determinant of cellular response and can regulate the proliferation, morphology and genetic expression of endothelial cells. In this study, the distribution of WSS under LVAD was quite different from that under normal conditions. Compared with the case of heart failure, the highest WSS region in the case of LVAD was mainly located in the ascending aorta and relatively uniform (Figure 7B).

It has been shown that because low WSS in the endothelium significantly increases the rate of mitosis or apoptosis, it is easier to induce leaky junctions at the stationary point to form more glue-linked cells, which provides a pathway for LDL accumulation in the arterial wall. Since the development and progression of diseases such as atherosclerosis and heart failure involve the accumulation of large amounts of LDL in the arterial wall, LVAD provided higher WSS at the cannula-ascending aortic junction and at the aortic arch, attenuating the extent of LDL accumulation. In addition, high WSS helps to increase the mechanical properties of the intima to withstand the mechanical force of the blood fluid, and the higher mechanical force may trigger plaque rupture and reduce the degree of atherosclerosis. However, the WSS values in the LVAD case were higher at the cannula-ascending aortic junction and at the aortic arch than in the healthy human ventricle, and in future medical studies, it needs to be sought whether it may have other effects (Xu, 2009).

### 3.2 Vortex identification

**FIGURE 8:**
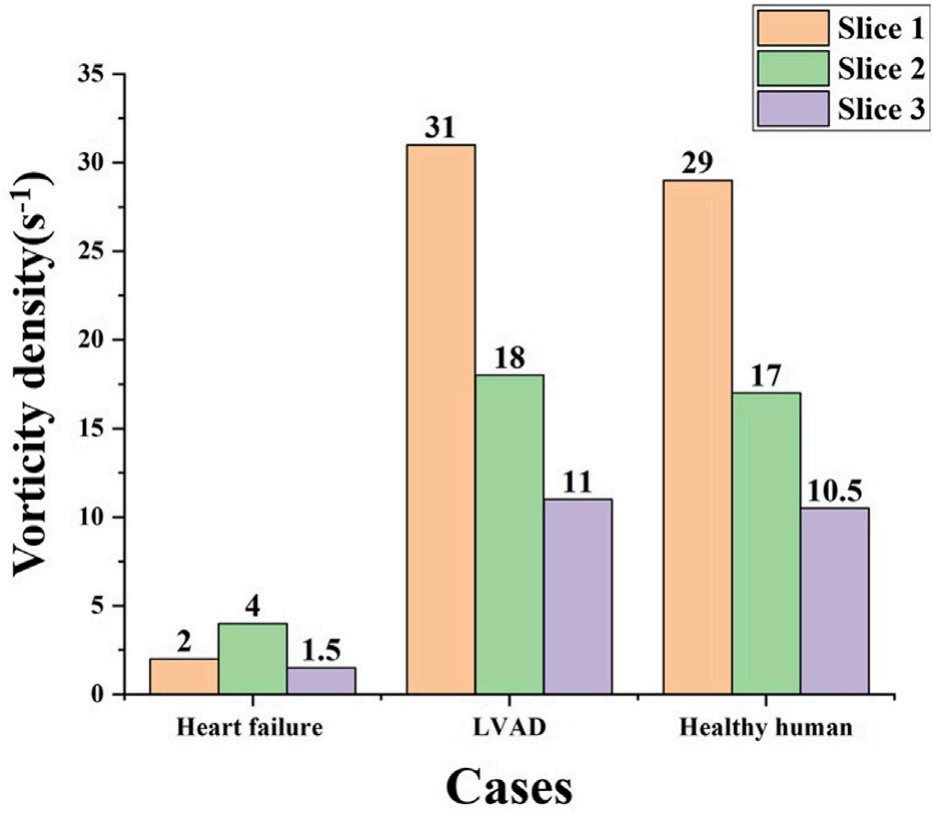
Longitudinal changes of area mean vorticity density along the central line of the aorta.

In order to further study the differences in aortic vorticity flow’s characteristics, the above three representative sections S1, S2 and S3 were continued to be used, and the changes in the area mean vorticity density (s^-1^) of the three sections of heart failure patients, LVAD patients and healthy human were summarized into a bar graph, as shown in Figure 8. The results showed that the overall vorticity density of the patients with heart failure was low. However, the vorticity density measured by LVAD in the three sections was much higher than that in patients with heart failure. The vorticity density decreased gradually along the central line of the aorta, and the vorticity density in the ascending aorta above the anastomosis was much higher than that in patients with heart failure, showing the maximum value. The vorticity density after LVAD intubation was similar to the normal ventricular values. Slightly higher than normal ventricle vorticity density.

**FIGURE 9:**
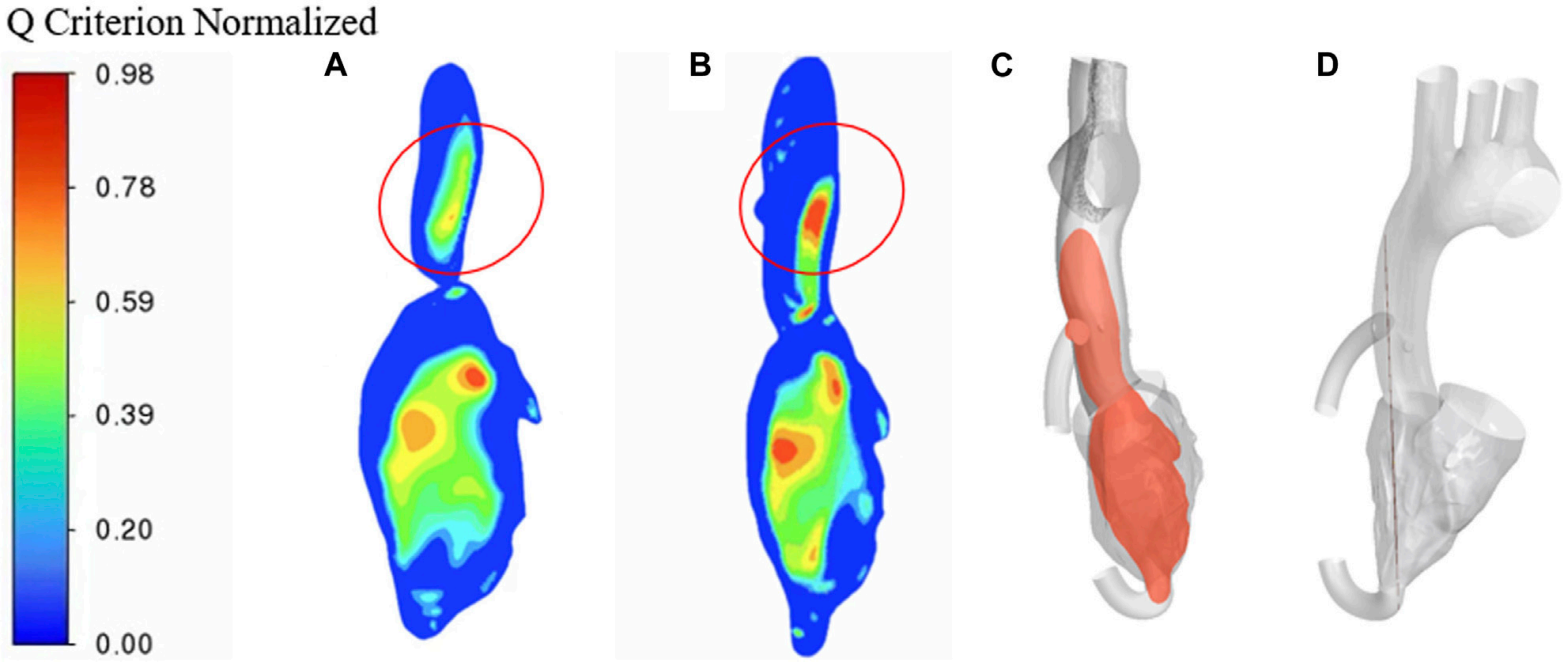
Q criterion cloud contour **(A)** heart failure case **(B)** LVAD case **(C)** Cross-sectional side view **(D)** Cross-sectional main view.

To better compare the vorticity flow generation in heart failure patients and LVAD case, here we take longitudinal slices of the whole ventricle model. So we gat Figure 9 which shows the cloud contour of Q criterion for the calculation cases of heart failure patients and LVAD. The red circle is near the junction. It is obviously found that the Q criterion of the LVAD case is much higher than that of the heart failure case. The maximum Q criterion of LVAD case reached 0.98, and it was close to the ascending aorta wall, indicating that vorticity flow structure was generated in LVAD case. We found counterclockwise and clockwise vortices in the upper mid-left ventricle in both patients with heart failure and healthy cases’ vorticity flows, positioned approximately near the anterior mitral leaflet. The distribution of the two vortices with different directions of rotation in healthy vorticity flows was asymmetric and the intensity was not similar, whereas the distribution of the two vortices with different directions of rotation in heart failure patients was nearly symmetric and the intensity was similar. This difference in distribution may be related to the larger ventricular area and the value of vorticity flow volume in patients (Bermejo et al., 2014). The vorticity density values between the left ventricle to the aorta kept decreasing under different conditions due to the rapid flow of blood out of the left ventricle through the aortic valve. In the ascending aorta region, the LVAD pumps blood rapidly into the ascending aortic branches, thus producing a vorticity density higher than that of normal humans. Since the LVAD rotation direction is counterclockwise, the same counterclockwise direction is produced here under the LVAD arithmetic, which is the same as but slightly higher than that produced in normal humans, and since the counterclockwise vorticity flow is a little further away from the ascending aorta, it exists for a longer period of time and thus can better maintain the LVAD effect (Ko et al., 2003). The vorticity intensity at the three sections of the ascending aorta can be seen that the vorticity flow intensity in the three sections of heart failure patients under the action of LVAD is significantly enhanced and higher than that in healthy human cases. The vorticity flow shape was larger and more rounded, indicating that LVAD can play a stable maintenance effect, and under the action of the vorticity flow, it is more difficult for atherosclerotic plaque and low-density lipoprotein to be deposited on the ascending aortic wall, which is beneficial to the treatment of heart failure patients with LVAD.

## 4 Limitations

It has been shown that steady-state simulations can provide more accurate hemodynamic parameter profiles and have practical medical value than transient boundary condition pulse simulations, which are closer to realistic situations. In the future, we can consider non-steady-state CFD simulations that are closer to the medical reality. In addition, the receiver position of LVAD may affect the effect on aortic hemodynamic parameters in actual medical work, and in future studies, we can consider changing the incision angle of receiver access to find the best intervention position. In addition, in this work, we used a zero-pressure boundary condition, and this has little effect on the accuracy of the results. In the future, we can consider using the flow-solid coupling method to study the effect of LVAD multi-Domain model on the hemodynamic parameters more closely to the actual medical situation.

## 5 Conclusion

We used CFD steady-state multi-Domain simulation to evaluate the difference in hemodynamic parameters of LVAD connected by catheter between the left ventricular apex and the ascending aorta and those of patients with heart failure. The steady-state simulation can provide more accurate hemodynamic parameters and has practical medical value. We proposed to construct a multi-Domain ventricular model and evaluated the effect of LVAD on hemodynamic parameters in a heart failure patient model by steady-state CFD numerical simulation. Using method Q criterion to demonstrate the generation of vorticity flow, and then we compared the vorticity density to determine the effect of LVAD. Compared with the LVAD case, heart failure case and the healthy human case, we found the following conclusions. Vorticity flow was formed under the action of LVAD high-speed jets, and the formation of vorticity flow helped to reduce the low-flow velocity zone and stagnation zone within the blood chamber. LVAD provided higher WSS at the cannula-ascending aortic junction and at the aortic arch. The vorticity density after LVAD intubation was similar to the normal ventricular values. Slightly higher than normal ventricle vorticity density. The vorticity flow shape was larger and more rounded. The above findings indicate that the use of LVAD for heart failure patients can attenuate the extent of LDL accumulation. Improving the intravascular surface flushing effect and reducing the risk of thrombus formation.

## Data Availability

The original contributions presented in the study are included in the article/Supplementary Material, further inquiries can be directed to the corresponding author.
